# Supplementary material for: Distinct Denitrification Phenotypes in Closely Related Bacteria: Clues to Understanding Variations in Nitrite Accumulation Among Stutzerimonas Strains
Source: Environ Microbiol. 2026 Apr 16;28(4):e70275. doi: 10.1111/1462-2920.70275 (PMC13086519; doi:10.1111/1462-2920.70275)
Supplement: Supplementary file 1 — Figure S1: Denitrification kinetics and electron flow rates in FNA strains during and after the transition from aerobic respiration to denitrification. Figure S2: Denitrification kinetics and electron flow rates in PNA strains during and after the transition from aerobic respiration to denitrification. Figure S3: Denitrification kinetics and electron flow rates in NNA strains during and after the transition from aerobic respiration to denitrification. Figure S5: Maximum likelihood phylogeny of full‐length NirS amino acid sequences in Stutzerimonas strains and gene arrangement of the nirS gene clusters. Figure S6: Denitrification kinetics (a), denitrification gene transcription (b), and electron flow rates to reductases (c) during and after the transition from aerobic respiration to denitrification for the NNA strain DSM 50238. Figure S7: Competition for electrons between NO3 − and NO2 − reduction pathways in the three Stutzerimonas strains, each representing the three groups of nitrite accumulators. Figure S8: Anaerobic nitrite accumulation kinetics of Stutzerimonas perfectomarina ZoBell sRNA overexpression mutants. Figure S9: MAFFT multiple sequence alignment of the intergenic region upstream of dnrE in 11 Stutzerimonas strains. Table S1: Description of the Stutzerimonas strains used in this study. Table S2: Primers used in this study. Table S3: Bacterial strains and plasmids used for generating Stutzerimonas decontaminans deletion mutant and the Stutzerimonas perfectomarina overexpression mutants in this study. [file EMI-28-e70275-s001.docx]

Supplementary information

# Distinct denitrification phenotypes in closely related bacteria: clues to understanding variations in nitrite accumulation among *Stutzerimonas* strains

Martin Menestreau^1^, Daniel A. Milligan^1^, Louise B. Sennett^1^, Linda Bergaust^1^, Lars R. Bakken^1^, Gary Rowley^2^, Morten Kjos^1^, James P. Shapleigh^3^ and Åsa Frostegård^1^

^1^ Faculty of Chemistry, Biotechnology, and Food Sciences, Norwegian University of Life Sciences, Norwegian University of Life Sciences, Ås, Norway

^2^ School of Biological Sciences, University of East Anglia, Norwich, United Kingdom

^3^ Department of Microbiology, Cornell University, Ithaca, NY, USA

Correspondance: Åsa Frostegård (asa.frostegard@nmbu.no)

Supplementary Figures

Figure S1. Denitrification kinetics and electron flow rates in FNA strains during and after the transition from aerobic respiration to denitrification

Figure S2. Denitrification kinetics and electron flow rates in PNA strains during and after the transition from aerobic respiration to denitrification

Figure S3. Denitrification kinetics and electron flow rates in NNA strains during and after the transition from aerobic respiration to denitrification

Figure S4. See separate file.

Figure S5. Maximum likelihood phylogeny of full-length NirS amino acid sequences in *Stutzerimonas* strains and gene arrangement of the *nirS* gene clusters

Figure S6. Denitrification kinetics (a), denitrification gene transcription (b), and electron flow rates to reductases (c) during and after the transition from aerobic respiration to denitrification for the NNA strain DSM 50238

Figure S7. Competition for electrons between NO_3_^-^ and NO_2_^-^ reduction pathways in the three Stutzerimonas strains, each representing the three groups of nitrite accumulators

Figure S8. Anaerobic nitrite accumulation kinetics of *Stutzerimonas perfectomarina* ZoBell sRNA overexpression mutants

Figure S9. MAFFT multiple sequence alignment of the intergenic region upstream of *dnrE* in 11 Stutzerimonas strains

Supplementary Tables

Table S1. Description of the *Stutzerimonas* strains used in this study.

Table S2. Primers used in this study.

Table S3. Bacterial strains and plasmids used for generating *Stutzerimonas decontaminans* deletion mutant and the *Stutzerimonas perfectomarina* overexpression mutants in this study.


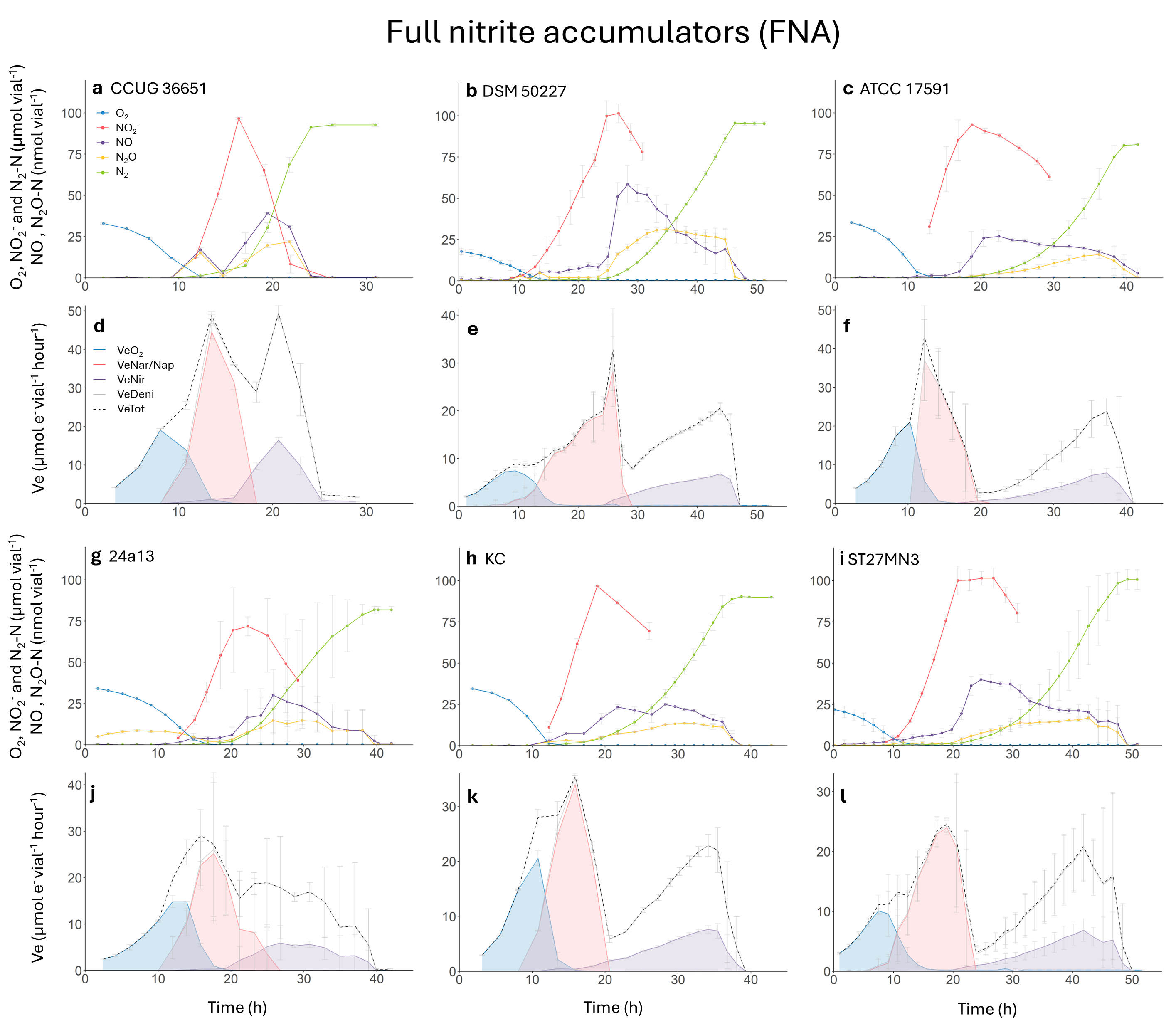


**Figure S1.** **Denitrification kinetics and electron flow rates in FNA strains during and after the transition from aerobic respiration to denitrification**. Results for six *Stutzerimonas* strains representing the FNA (full nitrite accumulator) group. The strains were incubated at 20 °C in 120 mL medical vials containing 50 mL Sistrom’s mineral medium supplemented with KNO_3_^-^ to an initial concentration of 2 mM (100 µmol vial⁻¹), with He and 1% O_2_ in the headspace. For each strain, the upper panel shows the measured amounts of each gas, and the lower pane shows the calculated rates of electron flow to O_2_ (blue), to NO_3_^-^ (red), and to NO_2_^-^ (purple). The dashed lines show the total electron flow. All graphs show mean values from three independent replicates (n = 3); error bars indicate standard deviation.

**
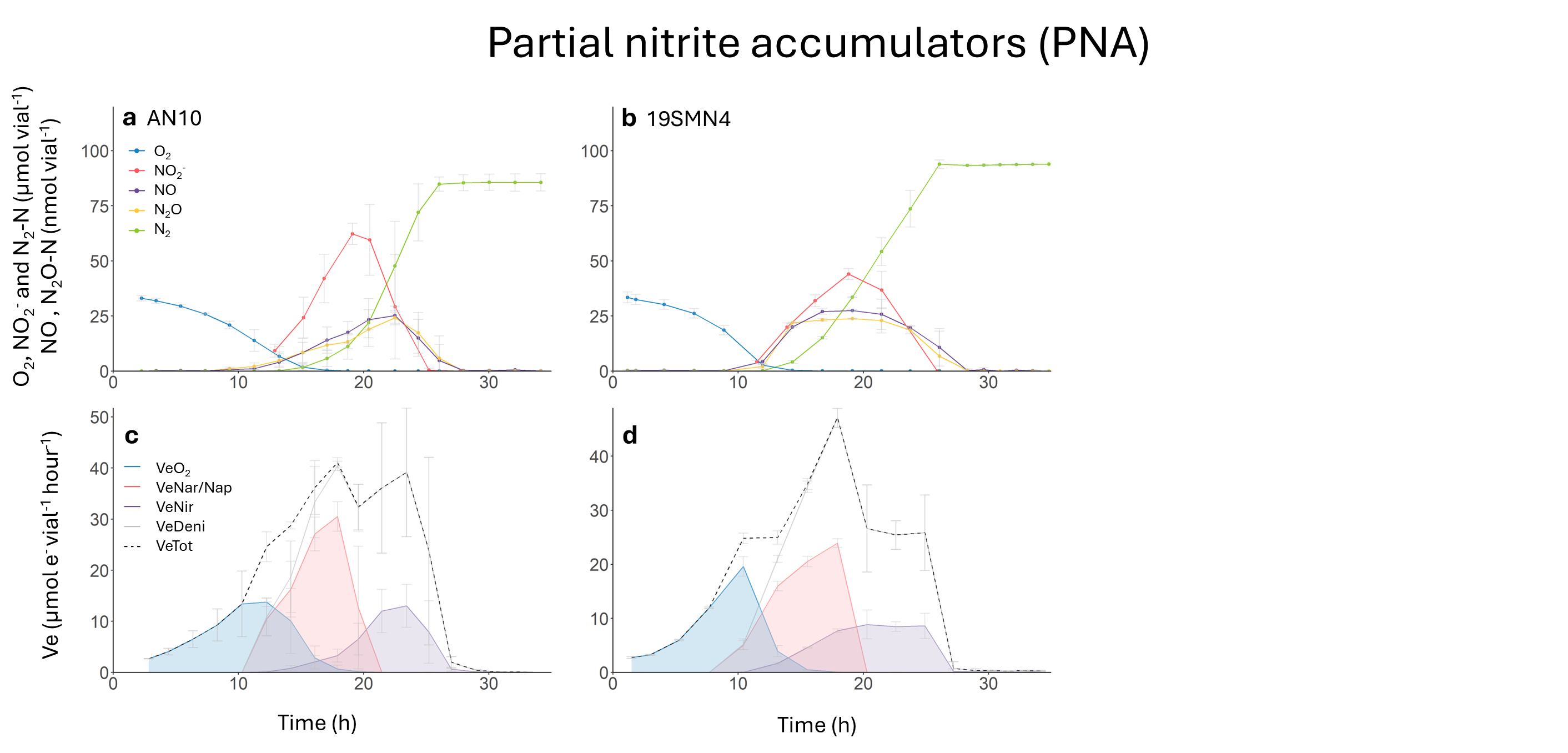
**

**Figure S2.** **Denitrification kinetics and electron flow rates in PNA strains during and after the transition from aerobic respiration to denitrification** Results for two *Stutzerimonas* strains representing the PNA (partial nitrite accumulator) group. The strains were incubated at 20 °C in 120 mL medical vials containing 50 mL Sistrom’s mineral medium supplemented with KNO_3_^-^ to an initial concentration of 2 mM (100 µmol vial⁻¹), with He and 1% O_2_ in the headspace. The shaded areas in panels c–d represent electron flow to O_2_ (blue), NO_3_^-^ (red), and NO_2_^-^ (purple). All graphs show mean values from three independent replicates (n = 3); error bars indicate standard deviation. The denitrification kinetics of *Stutzerimonas decontaminans* 19SMN4 were characterized in two independent experiments (see Fig. 4), both of which exhibited identical phenotypes.


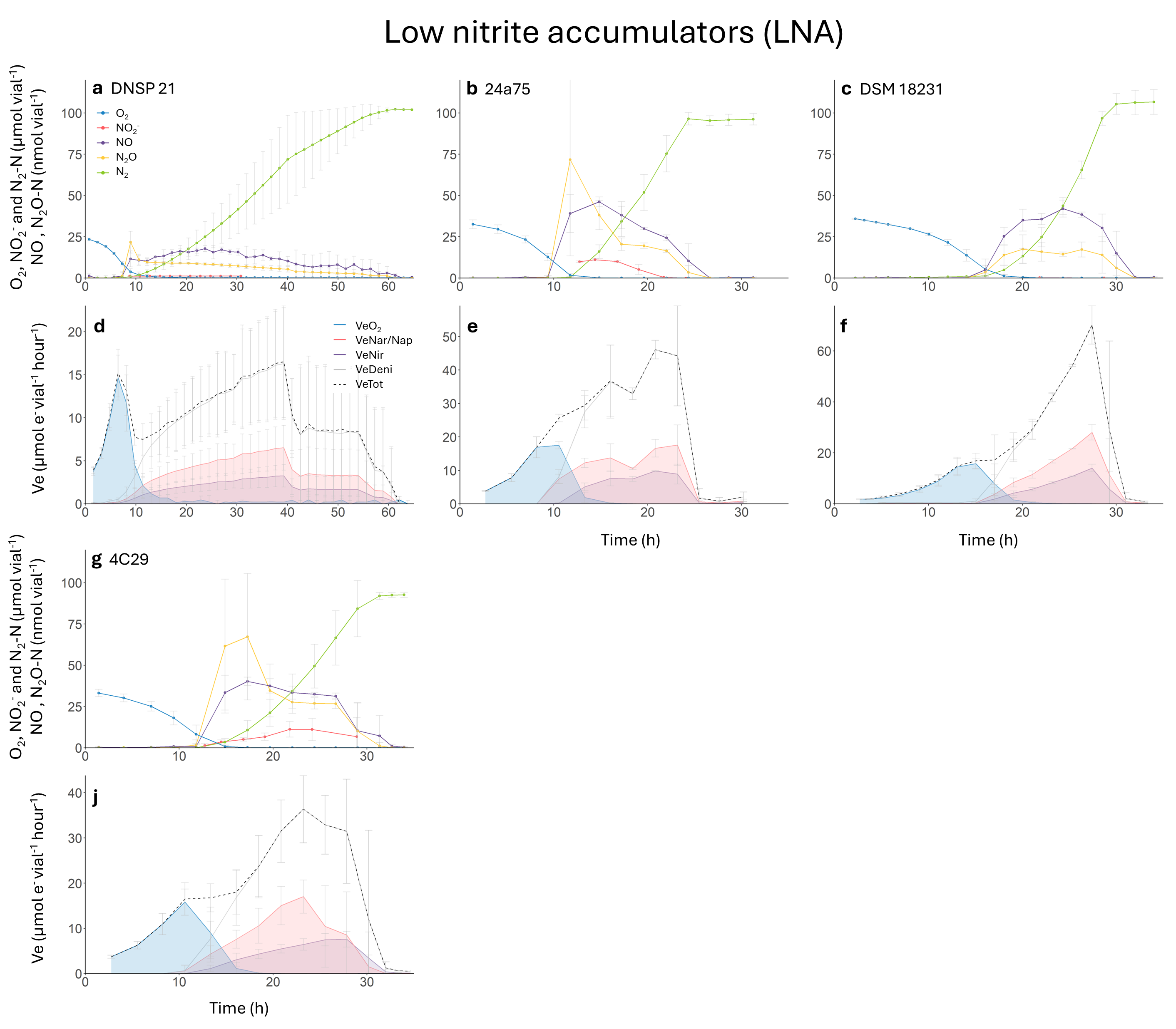


**Figure S3.** **Denitrification kinetics and electron flow rates in LNA strains during and after the transition from aerobic respiration to denitrification.** Results for five *Stutzerimonas* strains representing the LNA (low nitrite accumulator) group. The strains were incubated at 20 °C in 120 mL medical vials containing 50 mL Sistrom’s mineral medium supplemented with KNO_3_^-^ to an initial concentration of 2 mM (100 µmol vial⁻¹), with He and 1% O_2_ in the headspace. The shaded areas in panels d–f and j represent electron flow to O_2_ (blue), NO_3_^-^ (red), and NO_2_^-^ (purple). All graphs show mean values from three independent replicates (n = 3); error bars indicate standard deviation.

**
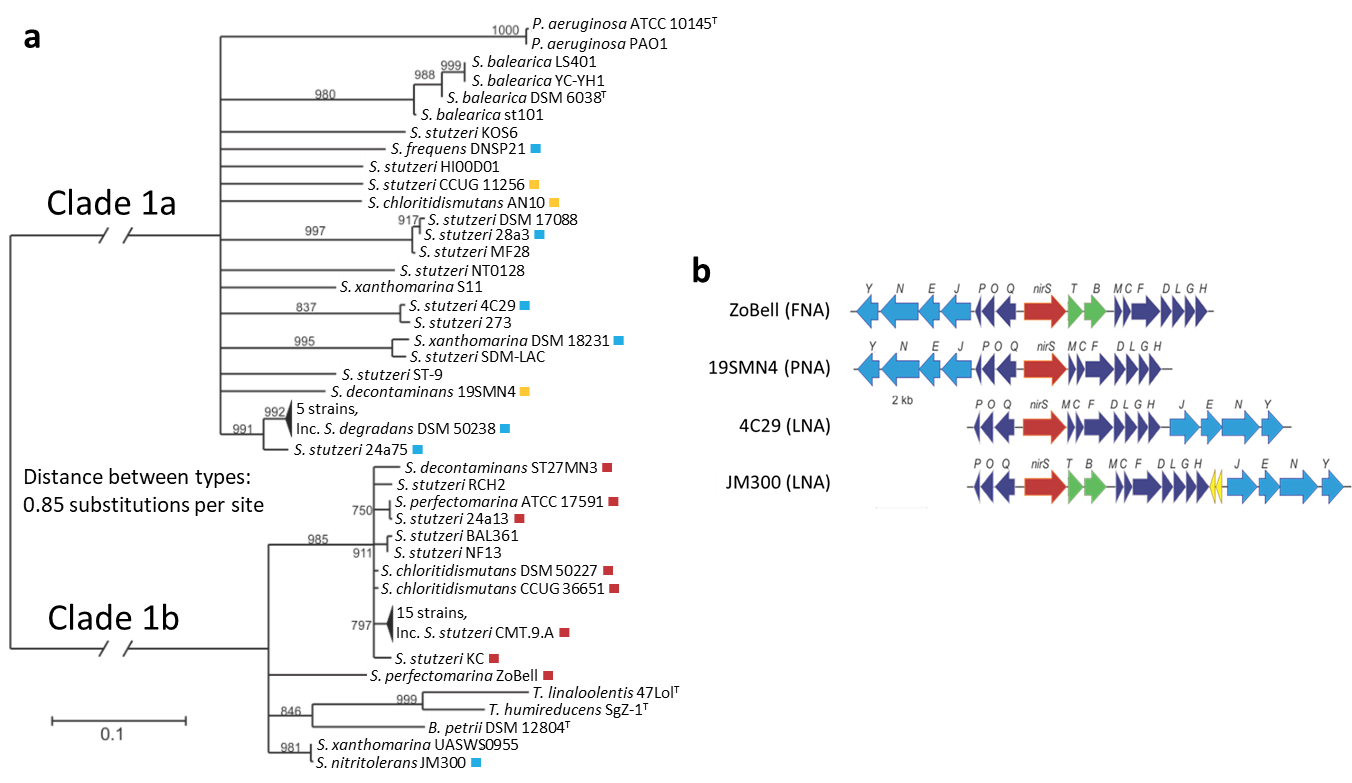
**

**Figure S5.** **Maximum likelihood phylogeny of full-length NirS amino acid sequences in *Stutzerimonas* strains and gene arrangement of the *nirS* gene clusters.** Only branches with bootstrap values ≥ 700 (of 1000) are shown. Strains are separated into two NirS types, termed clade 1a and clade 1b, according to the classification by Pold et al. (2024). Red, yellow, and blue indicate the three NO_2_^-^ accumulation phenotypes: FNA, PNA, and LNA, respectively. Panel b shows representative arrangements of the *nirS* gene cluster identified among the 18 studied *Stutzerimonas* strains, illustrated using four strains corresponding to the three NO_2_^-^ accumulation phenotypes: FNA (ZoBell), PNA (19SMN4), and LNA (4C29 and JM300). Uncharacterized ORFs with non-conserved synteny are shown in yellow.


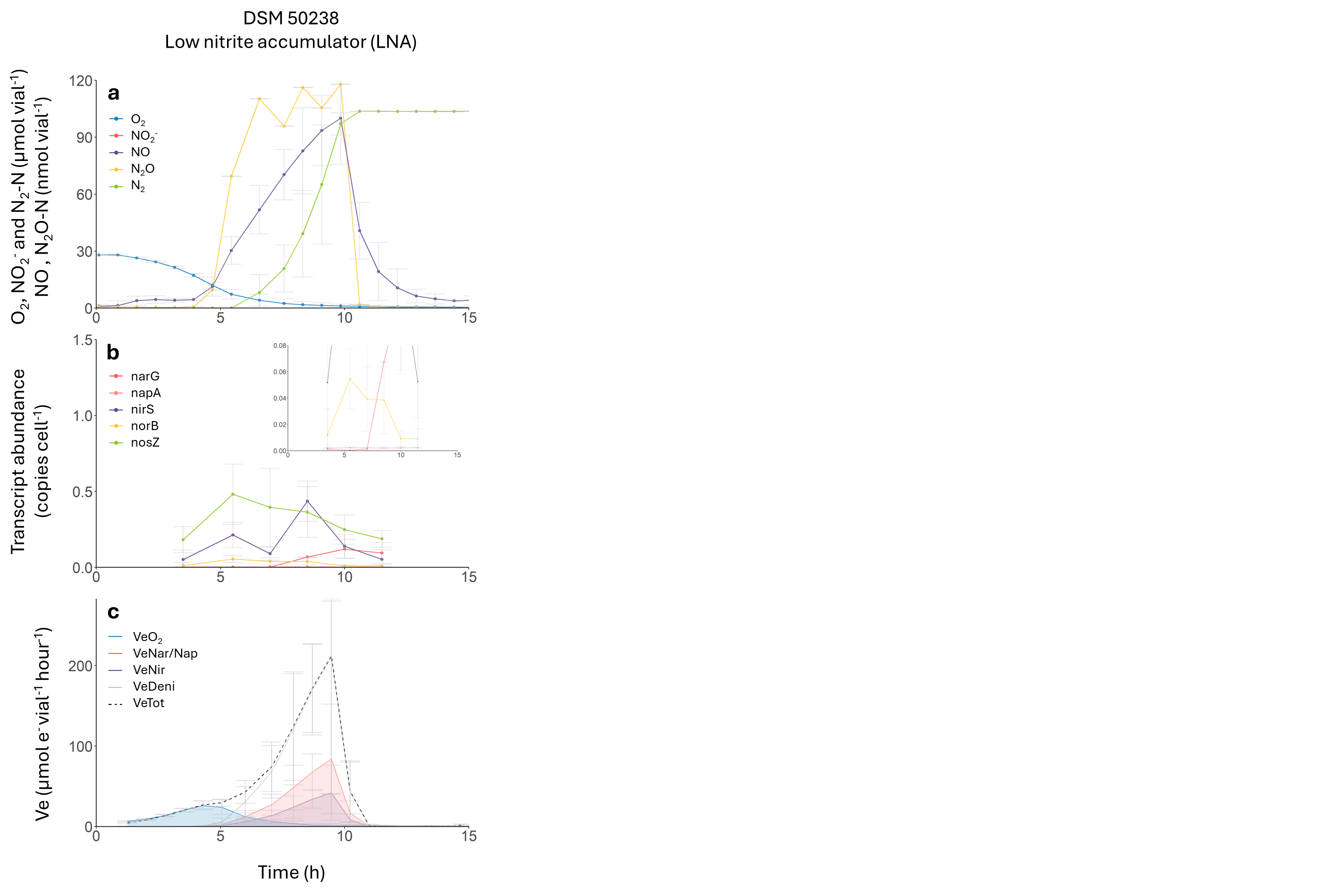


**Figure S6.** **Denitrification kinetics (a), denitrification gene transcription (b), and electron flow rates to reductases (c) during and after the transition from aerobic respiration to denitrification for the LNA strain DSM 50238.** The strain was incubated at 20 °C in 120 mL medical vials containing 50 mL Sistrom’s mineral medium supplemented with KNO_3_^-^ to an initial concentration of 2 mM (100 µmol vial⁻¹), with He and 1% O_2_ in the headspace. Graphs show mean values from three independent replicates (n = 3); error bars indicate standard deviation.


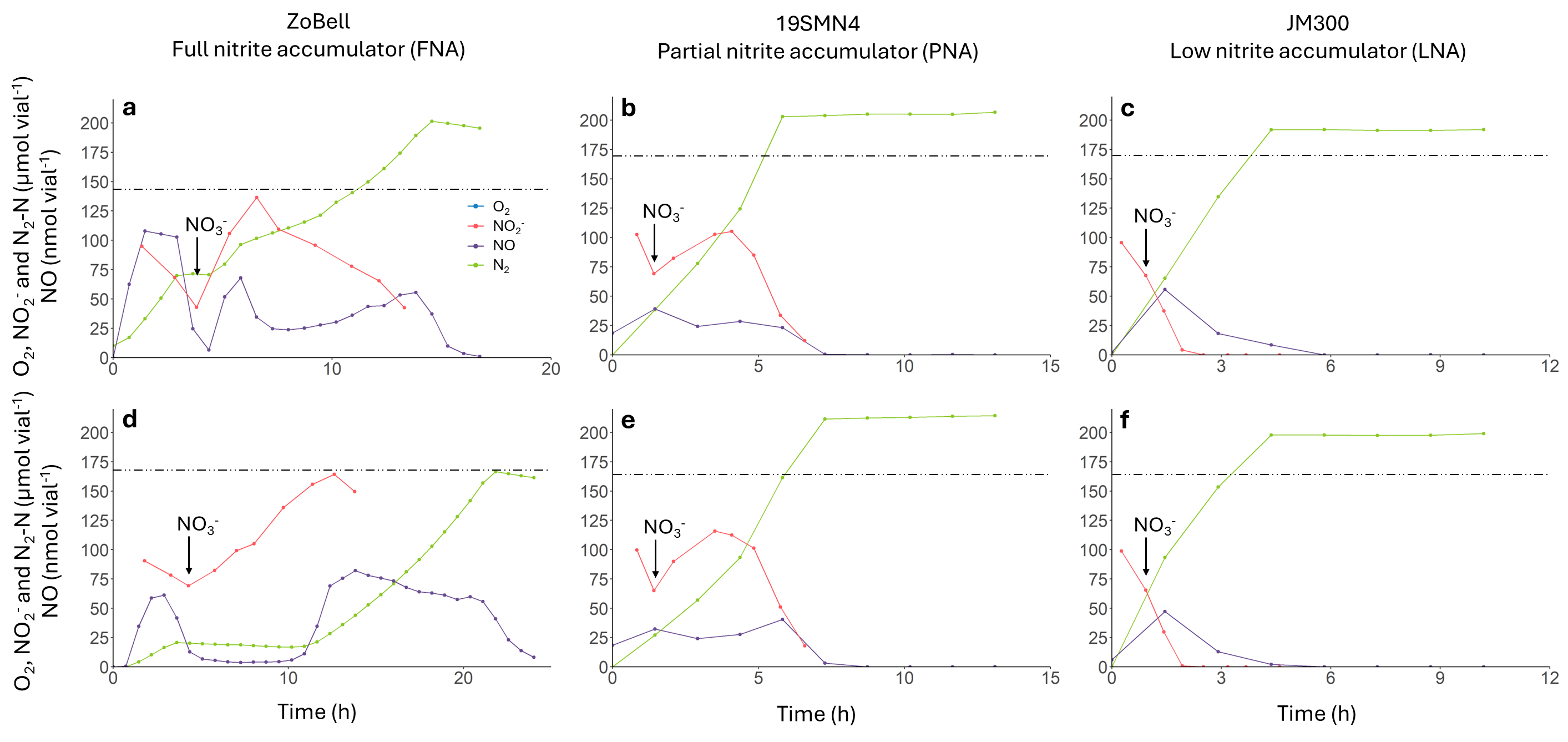


**Figure S7.** **Competition for electrons between NO_3_^-^ and NO_2_^-^ reduction pathways in three *Stutzerimonas* strains, each representing the three groups of nitrite accumulators**. The timing of NO_3_^-^ addition varied between replicate vials; one representative replicate is shown in Fig. 5a–c of the main text, and the two others are shown here. Cultures were first incubated under anoxic conditions (He headspace) in Sistrom´s medium containing NO_3_^-^, to produce cells with a complete denitrification proteome. At t = 0, cells from these precultures were transferred to fresh anoxic vials containing Sistrom’s medium with 2 mM NO_2_^-^ (100 µmol vial⁻¹), with no NO_3_^-^ initially present. When approximately one third of the NO_2_^-^ had been reduced, 100 µmol NO_3_^-^ (corresponding to 2 mM in the medium) was added (indicated by the arrow). Dashed lines represent the theoretical maximum accumulation of NO_2_^-^.

**
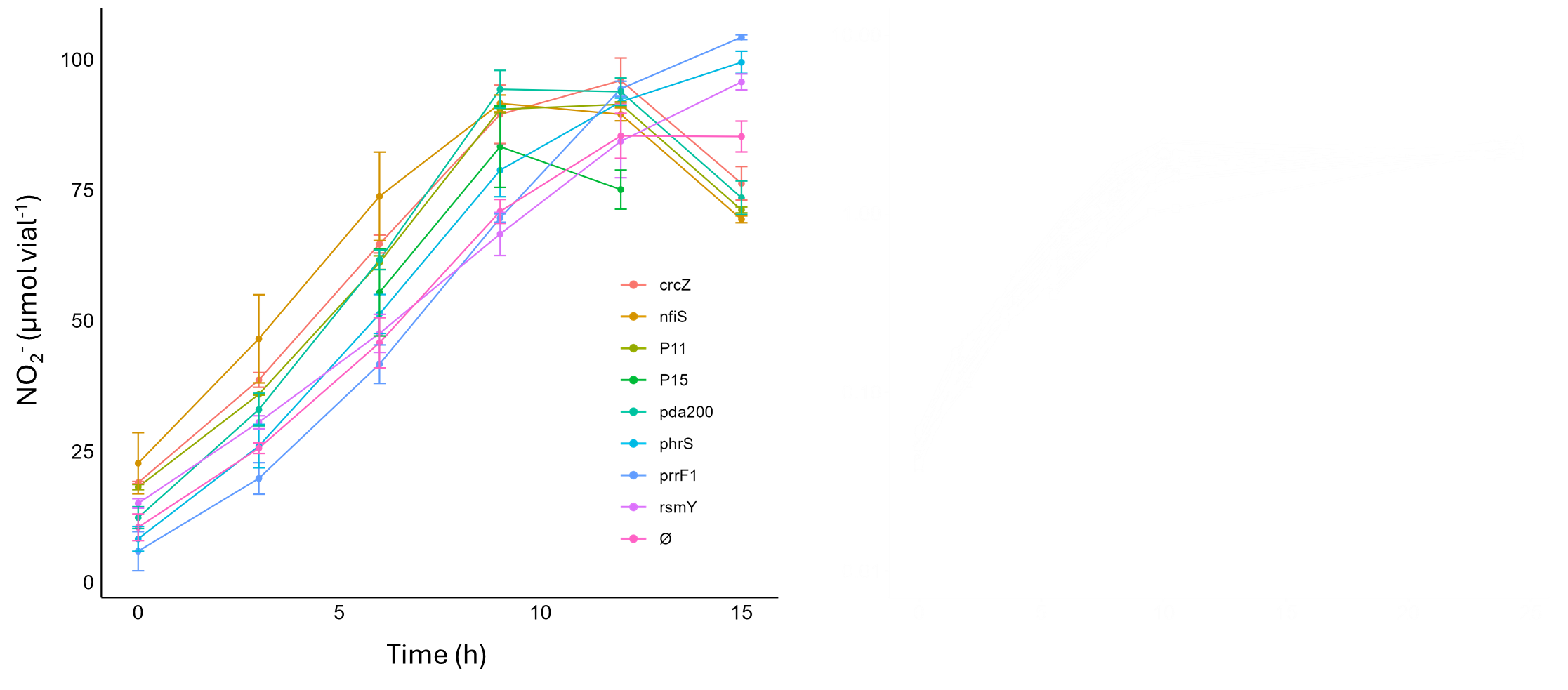
**

**Figure S8.** **Anaerobic nitrite accumulation kinetics of *Stutzerimonas perfectomarina* ZoBell sRNA overexpression mutants.** Eight sRNAs (crcZ, nfiS, P11, P15, pda200, phrS, prrF1, rsmY) (Lin et al., 2015; Zhan et al., 2016; Wang et al., 2022) were cloned into the arabinose-inducible vector pL2020 and transformed into *S. perfectomarina* ZoBell. Anaerobic cultures were grown in helium-flushed, sealed vials containing Sistrom’s medium supplemented with 30 µg mL^-1^ chloramphenicol and 2 mM nitrate (100 µmol vial^-1^) as the terminal electron acceptor. The cultures were induced by adding arabinose (2 mg mL^-1^) at the beginning of the incubation. Ø represent the *Stutzerimonas perfectomarina* Zobell strain carrying the empty pL2020 vector. Graph shows mean values from three independent replicates (n = 3); error bars indicate standard deviation.


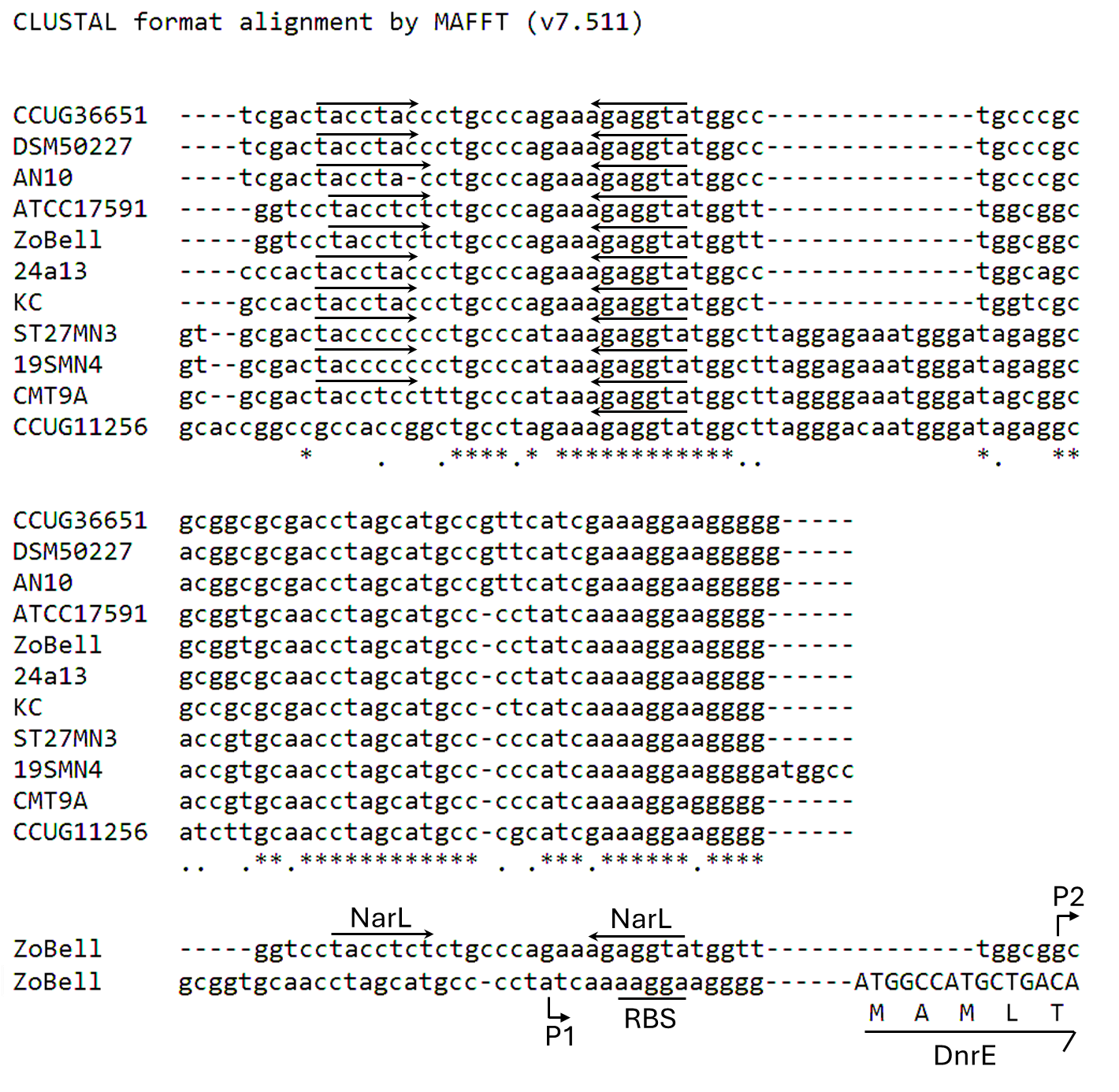


**Figure S9. MAFFT multiple sequence alignment of the intergenic region upstream of *dnrE* in 11 Stutzerimonas strains exhibiting the FNA and PNA phenotypes, all of which carry the dnrE gene.** Potential NarL binding sites are indicated by arrows, with the recognition heptamer consensus sequence in *E. coli* (TACYYMT), where Y represents T or C and M represents C or A (Härtig et al. 1999). Promoters are shown as cornered arrows, with P1 being NO_3_^-^ responsive (Vollack et al. 1999). Ribosome binding sites (RBS) are underlined. The *dnrE* coding sequence is shown in uppercase letters, and the corresponding translated amino acids are indicated above the half arrow.

**Table S1. Description of the *Stutzerimonas* strains used in this study.**

| Group | Strain | Old nomenclature | Genomovar | NCBI RefSeq | Isolation place and environment | References |
| --- | --- | --- | --- | --- | --- | --- |
| FNA | *S. chloritidismutans* | *P. stutzeri* | 3 | GCF_002890855.1 | Sweden; Water, borehole | Mulet et al., 2008 |
|  | CCUG 36651 * | CCUG 36651 |  |  |  |  |
|  | *S. chloritidismutans* | *P. stutzeri* | 3 | GCF_002843895.1 | Unknown; Clinical | Van Niel and Allen, 1952 |
|  | DSM 50227 * | DSM 50227 |  |  |  |  |
|  | *S. perfectomarina* | *P. stutzeri* | 2 | GCF_002890835.1 | Denmark; Clinical | Stanier et al., 1966 |
|  | ATCC 17591 * | ATCC 17591 |  |  |  |  |
|  | *S. perfectomarina* | *P. stutzeri* | 2 | GCF_000237885.1 | Pacific Ocean; Marine | ZoBell and Upham, 1944 |
|  | Zobell | ZoBell |  |  |  | Peña et al., 2012 |
|  | *S. stutzeri* | *P. stutzeri* | 16 | GCF_002909485.1 | Germany; Mineral oil-contaminated soil | Sikorski et al., 2002 |
|  | 24a13 * | 24a13 |  |  |  |  |
|  | *S. stutzeri* | *P. stutzeri* | 9 | GCF_002890795.1 | USA California; Aquifer | Sepúlveda-Torres et al., 2001 |
|  | KC * | KC |  |  |  |  |
|  | *S. decontaminans* | *P. stutzeri* | 4 | GCF_002890955.1 | Spain; Marine | Scotta et al., 2012 |
|  | ST27MN3 * | ST27MN3 |  |  |  |  |
|  | *S. stutzeri* | *P. stutzeri* | 1 | GCF_000195105.1 | Germany; Sorghastrum nutans rhizosphere | Krotzky and Werner, 1987 |
|  | CMT.9.A | CMT.9.A |  |  |  |  |
| PNA | *S. chloritidismutans* | *P. stutzeri* | 3 | GCF_000267545.1 | Spain; Contaminated marine sediment | Bosch et al., 2000 |
|  | AN10 | AN10 |  |  |  |  |
|  | *S. chloritidismutans* | *P. stutzeri* | 4 | GCF_000661915.1 | Spain; Contaminated marine sediment | Rosselló et al., 1991 |
|  | 19SMN4 | 19SMN4 |  |  |  |  |
|  | *S. stutzeri* | *P. stutzeri* | 1 | GCF_000219605.1 | Unknown; Spinal fluid | Chen et al., 2011 |
|  | CCUG 11256 | CCUG 11256 |  |  |  |  |
| LNA | *S. frequens* | *P. stutzeri* | 5 | GCF_002890935.1 | Spain; Wastewater | Rosselló et al., 1991 |
|  | DNSP21 * | DNSP21 |  |  |  |  |
|  | *S. stutzeri* | *P. stutzeri* | 17 | GCF_002890915.1 | Germany; Mineral oil-contaminated soil | Sikorski et al., 2002 |
|  | 24a75 * | 24a75 |  |  |  |  |
|  | *S. degradans* | *P. stutzeri* | 7 | GCF_002891015.1 | USA; Soil | Stanier et al., 1966 |
|  | DSM 50238 * | DSM 50238 |  |  |  |  |
|  | *S. xanthomarina* | *P. xanthomarina* | n/a | GCF_900129835.1 | Japan; Marine ascidian | Romanenko et al., 2005 |
|  | DSM 18231 | DSM18231 |  |  |  |  |
|  | *S. stutzeri* | *P. stutzeri* | 15 | GCF_002890895.1 | Germany; Marine sediment | Sikorski et al., 2002 |
|  | 4C29 * | 4C29 |  |  |  |  |
|  | *S. stutzeri* | *P. stutzeri* | 14 | GCF_002890995.1 | Israel; Soil | Sikorski et al., 2002 |
|  | 28a3 * | 28a3 |  |  |  |  |
|  | *S. nitritolerans* | *P. stutzeri* | 8 | GCF_000279165.1 | USA; Soil | Rosselló-Mora et al., 1996 |
|  | JM300 | JM300 |  |  |  | Busquets et al., 2012 |

Strains marked with an asterisk (*) were sequenced as part of this study.

**Table S2. Primers used in this study.**

| Primer | Sequence 5’-3’ | Description |
| --- | --- | --- |
| Fw *narG* ZoBell | ATCGAGTGCTTCAACGCCAA | Targeting *narG* of the strain ZoBell for qPCR analysis |
| Rv *narG* ZoBell | ATCATGTGGGTGGGCTTGAG |  |
| Fw *narG* JM300 | GAAGGGCAAGAAGAGCAACG | Targeting *narG* of the strain JM300 for qPCR analysis |
| Rv *narG* JM300 | CAGGGTGAGCATGATCAGGT |  |
| Fw *napA* ZoBell | CGCCAACAAGCTGATCAACA | Targeting *napA* of the strain ZoBell and JM300 for qPCR analysis |
| Fw *napA* JM300 | TTTCTTCGACGCCAACAAGC |  |
| Rv *napA* ZoBell and JM300 | TCTTCACGGCGCATTTCTTG |  |
| Fw *nirS* ZoBell | CGTGGTATCGCTCATCTCCA | Targeting *nirS* of the strain ZoBell for qPCR analysis |
| Rv *nirS* ZoBell | GGTCTTGACGAACAGGTTGC |  |
| Fw *nirS* JM300 | TGGCAGCTCTGATCGATACC | Targeting *nirS* of the strain JM300 for qPCR analysis |
| Rv *nirS* JM300 | TGCTCCTTGTACTTGGCGTA |  |
| Fw *norB* ZoBell | CTGTTCGCGTTCTACAACCC | Targeting *norB* of the strain ZoBell for qPCR analysis |
| Rv *norB* ZoBell | GGCGATGATCACGTACAACC |  |
| Fw *norB* JM300 | TCCTGATCAAGATCACCGGC | Targeting *norB* of the strain JM300 for qPCR analysis |
| Rv *norB* JM300 | CAGCACCATGGCGAAGAAC |  |
| Fw *nosZ* ZoBell and JM300 | CGGTGTTCAACGTCGACTC | Targeting *nosZ* of the strain ZoBell and JM300 for qPCR analysis |
| Rv *nosZ* ZoBell and JM300 | TGTTGGCCTTGTCGTTGATG |  |
| Fw *narG* DSM 50238 | TACGTCGGTCAGGAAAAGC | Targeting *narG* of the strain DSM 50238 for qPCR analysis |
| Rv *narG* DSM 50238 | GCGAGCTGTGGTTGTAGAAG |  |
| Fw *napA* DSM 50238 | TTCTTCGACGCCAACAAAC | Targeting *napA* of the strain DSM 50238 for qPCR analysis |
| Rv *napA* DSM 50238 | TGCTGACCACTTCGATCTTG |  |
| Fw *nirS* DSM 50238 | TGTGGACAAGCAGGAATACC | Targeting *nirS* of the strain DSM 50238 for qPCR analysis |
| Rv *nirS* DSM 50238 | AGAATCTTGCCGGTTTCCT |  |
| Fw *norB* DSM 50238 | TTGACCGTGAAGTGATCGAG | Targeting *norB* of the strain DSM 50238 for qPCR analysis |
| Rv *norB* DSM 50238 | GGTGACCTGTACCGATGATG |  |
| Fw *nosZ* DSM 50238 | ACGCCGAGAAGATGGAAAT | Targeting *nosZ* of the strain DSM 50238 for qPCR analysis |
| Rv *nosZ* DSM 50238 | AAGGCCTTCTCCGAGTTGTA |  |
| Fw pCasPA | ATTATGTTGGTCCATTGGC | Specific primers of pCasPA |
| Rv pCasPA | CAACCAGTATAACGGCGAC |  |
| Fw pACRISPR | ATTAATCATCCGGCTCGTAT | Verify the insertion of sgRNA and repair template |
| Rv pACRISPR | TATGACCATGATTACGCCA |  |
| Fw sgRNA *narG* | GTGGGCGGCGCAACATGTCCCCAG | sgRNA sequence for deletion of *narG* |
| Rv sgRNA *narG* | AAACCTGGGGACATGTTGCGCCGC |  |
| Fw sgRNA *dnrE* | GTGGACGCAAGGTGTTGAGCACTG | sgRNA sequence for deletion of *dnrE* |
| Rv sgRNA *dnrE* | AAACCAGTGCTCAACACCTTGCGT |  |
| Fw up *narG* | TGTCCATACCCATGGTCTAGACCCGGTCGGCACCATCCA | Amplification for 1kb upstream repair of *narG* |
| Rv up *narG* | GGCCTATTCCTGGTTTCTCTCCTCACTCCGGT |  |
| Fw down *narG* | AGAGAAACCAGGAATAGGCCATGAAGATTCGTTC | Amplification for 1kb downstream repair of *narG* |
| Rv down *narG* | GGGAGTATGAAAAGTCTCGAGGTAGACCGGCGACTTCTGC |  |
| Fw up *dnrE* | TGTCCATACCCATGGTCTAGATGCCGAGGACATGCTCGT | Amplification for 1kb upstream repair of *dnrE* |
| Rv up *dnrE* | AAGGAAGGGGGCCCTACTCGGACGGATCG |  |
| Fw down *dnrE* | CGAGTAGGGCCCCCTTCCTTTTGATGGGGGC | Amplification for 1kb downstream repair of *dnrE* |
| Rv down *dnrE* | GGGAGTATGAAAAGTCTCGAGACGGCGGTAAGCCCTTGAC |  |
| Fw pL2020 PrrF1 | AGGAGGAATTACATAGAGAATTGTTATTATTATCGCAACT | Amplification for the sRNA PrrF1 |
| Rv pL2020 PrrF1 | CAAAACAGCCAAGCTCAGGCCGATTACGTCTGGTACA |  |
| Fw pL2020 PhrS | AGGAGGAATTACATATTGCCGGTTTAACTTGAACCC | Amplification for the sRNA PhrS |
| Rv pL2020 PhrS | CAAAACAGCCAAGCTGATCAGTCGCCTGGCCAC |  |
| Fw pL2020 RsmY | AGGAGGAATTACATAACGGTCTGGCGGTAATCTACTG | Amplification for the sRNA RsmY |
| Rv pL2020 RsmY | CAAAACAGCCAAGCTGGCGAAGGTCATTTGCTTCATCG |  |
| Fw pL2020 CrcZ | AGGAGGAATTACATAACGACAACTGCTTACTTAATGGC | Amplification for the sRNA CrcZ |
| Rv pL2020 CrcZ | CAAAACAGCCAAGCTGTGTGGCGGAATGACGGG |  |
| Fw pL2020 P11 | AGGAGGAATTACATATTCGCACCATGAGGGCGC | Amplification for the sRNA P11 |
| Rv pL2020 P11 | CAAAACAGCCAAGCTATGCCGTTGCCGATCATCACC |  |
| Fw pL2020 P15 | AGGAGGAATTACATATCGCCATGCGGGTTGAAA | Amplification for the sRNA P15 |
| Rv pL2020 P15 | CAAAACAGCCAAGCTGGCGTCGAAGCGGCTAAA |  |
| Fw pL2020 NfiS | AGGAGGAATTACATAGCGCCGCCAGTCCCACCGAA | Amplification for the sRNA NfiS |
| Rv pL2020 NfiS | CAAAACAGCCAAGCTTCGGGTAGCGCCGCGATTGA |  |
| Fw pL2020 pda200 | AGGAGGAATTACATACGCGCCCGGCCTTGATCG | Amplification for the sRNA pda200 |
| Rv pL2020 pda200 | CAAAACAGCCAAGCTTGCAGGCCGAACCGACCTTCATCG |  |
| Fw pL2020 paiI | AGGAGGAATTACATACGCGGGACTTAGTCTTGAGC | Amplification for the sRNA paiI |
| Rv pL2020 paiI | CAAAACAGCCAAGCTCAGGCACCCTTCGGTACTCG |  |

**Table S3. Bacterial strains and plasmids used for generating *Stutzerimonas decontaminans* deletion mutant and the *Stutzerimonas perfectomarina* overexpression mutants in this study.**

| Strain | Description | Source |
| --- | --- | --- |
| *E. coli* DH5α | Cloning strain | Lab stock |
| *S. decontaminans* 19SMN4 | Wild-type | DSM |
| 19SMN4 pCasPA | 19SMN4 harboring pCasPA | This study |
| 19SMN4 *ΔnarG* | 19SMN4 *narG* gene deleted | This study |
| 19SMN4 *ΔdnrE* | 19SMN4 *dnrE* gene deleted | This study |
| *S. perfectomarina* ZoBell | Wild-type | ATCC |
| ZoBell PrrF1 | ZoBell harboring pL2020 PrrF1 | This study |
| ZoBell PhrS | ZoBell harboring pL2020 PhrS | This study |
| ZoBell RsmY | ZoBell harboring pL2020 RsmY | This study |
| ZoBell CrcZ | ZoBell harboring pL2020 CrcZ | This study |
| ZoBell P11 | ZoBell harboring pL2020 P11 | This study |
| ZoBell P15 | ZoBell harboring pL2020 P15 | This study |
| ZoBell NfiS | ZoBell harboring pL2020 NfiS | This study |
| ZoBell pda200 | ZoBell harboring pL2020 pda200 | This study |
| ZoBell paiI | ZoBell harboring pL2020 paiI | This study |
| Plasmids | Description | Reference |
| pCasPA | Tetracyline resistant, for expression of Cas9 protein and λ-Red recombination system | (Chen et al., 2018) |
| pACRISPR | Carbenicillin resistant, for expression of sgRNA and assembling homologous repair arms | (Chen et al., 2018) |
| pACRISPR *ΔnarG* | pACRISPR derivative for *narG* deletion | This study |
| pACRISPR *ΔdnrE* | pACRISPR derivative for *dnrE* deletion | This study |
| pL2020 | Chloramphenicol resistant, araBAD based expression vector for inducible protein production | (Sommer et al.,2017) |
| pL2020 PrrF1 | pL2020 derivative carrying the sRNA PrrF1 | This study |
| pL2020 PhrS | pL2020 derivative carrying the sRNA PhrS | This study |
| pL2020 RsmY | pL2020 derivative carrying the sRNA RsmY | This study |
| pL2020 CrcZ | pL2020 derivative carrying the sRNA CrcZ | This study |
| pL2020 P11 | pL2020 derivative carrying the sRNA P11 | This study |
| pL2020 P15 | pL2020 derivative carrying the sRNA P15 | This study |
| pL2020 NfiS | pL2020 derivative carrying the sRNA NfiS | This study |
| pL2020 pda200 | pL2020 derivative carrying the sRNA pda200 | This study |
| pL2020 paiI | pL2020 derivative carrying the sRNA paiI | This study |

References:

Bosch, R., García-Valdés, E., & Moore, E. R. (2000). Complete nucleotide sequence and evolutionary significance of a chromosomally encoded naphthalene-degradation lower pathway from Pseudomonas stutzeri AN10. Gene, 245(1), 65–74. https://doi.org/10.1016/s0378-1119(00)00038-x

Busquets, A., Peña, A., Gomila, M., Bosch, R., Nogales, B., García-Valdés, E., Lalucat, J., & Bennasar, A. (2012). Genome sequence of Pseudomonas stutzeri strain JM300 (DSM 10701), a soil isolate and model organism for natural transformation. Journal of Bacteriology, 194(19), 5477–5478. https://doi.org/10.1128/JB.01257-12

Chen, M., Yan, Y., Zhang, W., Lu, W., Wang, J., Ping, S., & Lin, M. (2011). Complete genome sequence of the type strain Pseudomonas stutzeri CGMCC 1.1803. Journal of Bacteriology, 193(21), 6095. https://doi.org/10.1128/JB.06061-11

Chen, W., Zhang, Y., Zhang, Y., Pi, Y., Gu, T., Song, L., Wang, Y., & Ji, Q. (2018). CRISPR/Cas9-based genome editing in Pseudomonas aeruginosa and cytidine deaminase-mediated base editing in Pseudomonas species. iScience, 6, 222–231. https://doi.org/10.1016/j.isci.2018.07.024

Härtig, E., Schiek, U., Vollack, K.-U., & Zumft, W. G. (1999). Nitrate and Nitrite Control of Respiratory Nitrate Reduction in Denitrifying *Pseudomonas stutzeri* by a Two-Component Regulatory System Homologous to NarXL of *Escherichia coli*. *Journal of Bacteriology*, *181*(12), 3658–3665. https://doi.org/10.1128/JB.181.12.3658-3665.1999

Krotzky, A., & Werner, D. (1987). Nitrogen fixation in Pseudomonas stutzeri. Archives of Microbiology, 147, 48–57. https://doi.org/10.1007/BF00492904

Lees, J. A., Harris, S. R., Tonkin-Hill, G., Gladstone, R. A., Lo, S. W., Weiser, J. N., Corander, J., Bentley, S. D., & Croucher, N. J. (2019). Fast and flexible bacterial genomic epidemiology with PopPUNK. Genome Research, 29(2), 304–316. https://doi.org/10.1101/gr.241455.118

Lin, M., Yan, Y., Lu, W., Zhan, Y., Zhang, Y., & Elmerich, C. (2015). Regulatory coupling of nitrogen and carbon metabolism in nitrogen‐fixing Pseudomonas stutzeri A1501. In F. J. De Bruijn (Ed.), Biological nitrogen fixation (1st ed., pp. 109–120). Wiley. https://doi.org/10.1002/9781119053095.ch10

Letunic, I., & Bork, P. (2024). Interactive Tree of Life (iTOL) v6: Recent updates to the phylogenetic tree display and annotation tool. Nucleic Acids Research, 52(W1), W78–W82. https://doi.org/10.1093/nar/gkae268

Mulet, M., Gomila, M., Gruffaz, C., Meyer, J. M., Palleroni, N. J., Lalucat, J., & García-Valdés, E. (2008). Phylogenetic analysis and siderotyping as useful tools in the taxonomy of Pseudomonas stutzeri: Description of a novel genomovar. International Journal of Systematic and Evolutionary Microbiology, 58(Pt 10), 2309–2315. https://doi.org/10.1099/ijs.0.65797-0

Peña, A., et al. (2012). Draft genome of Pseudomonas stutzeri strain ZoBell (CCUG 16156), a marine isolate and model organism for denitrification studies. Journal of Bacteriology, 194(5), 1277–1278. https://doi.org/10.1128/JB.06648-11

Romanenko, L. A., Uchino, M., Falsen, E., Lysenko, A. M., Zhukova, N. V., & Mikhailov, V. V. (2005). Pseudomonas xanthomarina sp. nov., a novel bacterium isolated from marine ascidian. The Journal of General and Applied Microbiology, 51(2), 65–71. https://doi.org/10.2323/jgam.51.65

Rosselló, R., García-Valdés, E., Lalucat, J., & Ursing, J. (1991). Genotypic and phenotypic diversity of Pseudomonas stutzeri. Systematic and Applied Microbiology, 14, 150–157. https://doi.org/10.1016/S0723-2020(11)80294-8

Rosselló-Mora, R. A., Lalucat, J., & Moore, E. R. (1996). Strain JM300 represents a new genomovar within Pseudomonas stutzeri. Systematic and Applied Microbiology, 19, 596–599. https://doi.org/10.1016/S0723-2020(96)80031-2

Scotta, C., Mulet, M., Sánchez, D., Gomila, M., Ramírez, A., Bennasar, A., García-Valdés, E., Holmes, B., & Lalucat, J. (2012). Identification and genomovar assignation of clinical strains of Pseudomonas stutzeri. European Journal of Clinical Microbiology & Infectious Diseases, 31(9), 2133–2139. https://doi.org/10.1007/s10096-012-1547-4

Sepúlveda-Torres, L. C., Zhou, J., Guasp, C., Lalucat, J., Knaebel, D., Plank, J. L., & Criddle, C. S. (2001). Pseudomonas sp. strain KC represents a new genomovar within Pseudomonas stutzeri. International Journal of Systematic and Evolutionary Microbiology, 51(Pt 6), 2013–2019. https://doi.org/10.1099/00207713-51-6-2013

Sikorski, J., Möhle, M., & Wackernagel, W. (2002). Identification of complex composition, strong strain diversity and directional selection in local Pseudomonas stutzeri populations from marine sediment and soils. Environmental Microbiology, 4(8), 465–476. https://doi.org/10.1046/j.1462-2920.2002.00325.x

Sommer, M., Xie, H., & Michel, H. (2017). Pseudomonas stutzeri as an alternative host for membrane proteins. Microbial Cell Factories, 16(1), 157. https://doi.org/10.1186/s12934-017-0771-0

Van Niel, C. B., & Allen, M. B. (1952). A note on Pseudomonas stutzeri. Journal of Bacteriology, 64(3), 413–422. https://doi.org/10.1128/jb.64.3.413-422.1952

Vollack, K., Härtig, E., Körner, H., & Zumft, W. G. (1999). Multiple transcription factors of the FNR family in denitrifying *Pseudomonas stutzeri*: Characterization of four *fnr* ‐like genes, regulatory responses and cognate metabolic processes. *Molecular Microbiology*, *31*(6), 1681–1694. https://doi.org/10.1046/j.1365-2958.1999.01302.x

Wang, M., Liang, Y., Li, F., Shen, S., Huang, X., & Sun, Y. (2022). Enhancement of biological denitrification by the addition of novel sRNA Pda200 under antibiotic pressure. Bioresource Technology, 365, 128113. https://doi.org/10.1016/j.biortech.2022.128113

ZoBell, C. E., & Upham, H. C. (1944). A list of marine bacteria including description of sixty species. Bulletin of the Scripps Institution of Oceanography, 5, 239–292.
