# Supplementary material for: Distinct Denitrification Phenotypes in Closely Related Bacteria: Clues to Understanding Variations in Nitrite Accumulation Among Stutzerimonas Strains
Source: Environ Microbiol. 2026 Apr 16;28(4):e70275. doi: 10.1111/1462-2920.70275 (PMC13086519; doi:10.1111/1462-2920.70275)
Supplement: Supplementary file 2 — Figure S4: Stutzerimonas genome clustering tree and NO2 − accumulation phenotypes of the strains examined in this study. [file EMI-28-e70275-s002.pdf]

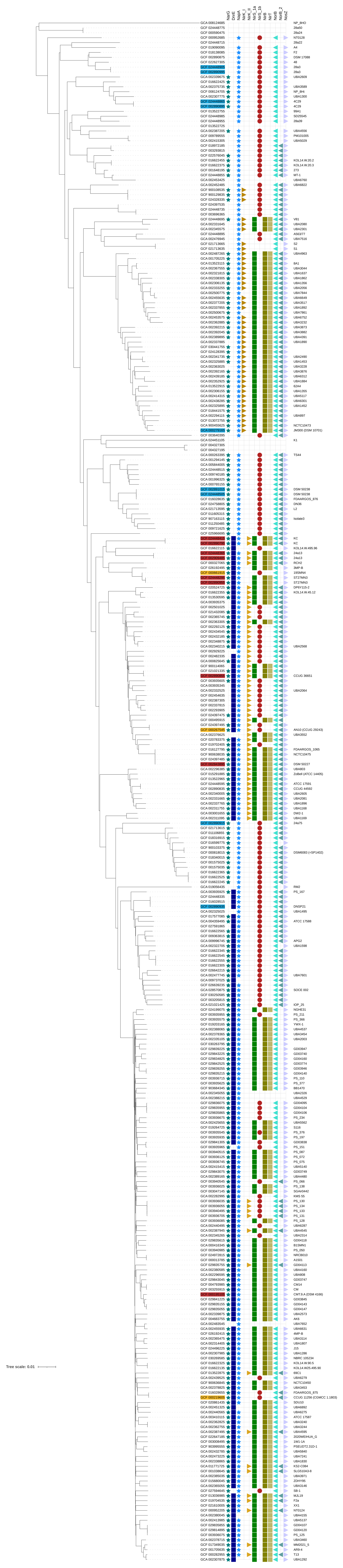

NarG
DnrE
NapA
NirK_I
NirK_II
NirS_1a
NirS_1b
NirB
NirT
NorB
NorB_2
NosZ
Tree scale: 0.01

Figure S4. *Stutzerimonas* genome clustering tree and NO_2^- accumulation phenotypes of the strains examined in this study. The three NO_2^- accumulation groups are indicated by different colors in the phylogenetic tree. Red: Full NO_2^- accumulators (FNA); Yellow: Partial NO_2^- accumulators (PNA); Blue: Low NO_2^- accumulators (LNA). Selected genes associated with denitrification are indicated. To generate the tree an extensive collection of genomes from organisms given the genus name *Stutzerimonas* was gathered. This was done using the advanced search in GTDB (https://gtdb.ecogenomic.org/). The search term was g__Stutzerimonas and the filters used were CheckM completeness > 95%, CheckM contamination < 10%, and number of contigs < 80. At the time this was run (October, 2024) this returned 322 genomes. After manual filtering to remove small genomes and genomes with other issues a final set of 314 genomes was downloaded from NCBI using the curl file generated by GTDB. Some strains have been sequenced multiple times, but these were not trimmed to a single genome, therefore, some strains appear more than once in the output. These genomes were clustered based on k-mer distances using PopPUNK 2.6.0 (POPulation Partitioning Using Nucleotide Kmers) (Lees et al., 2019). For creating the database these command line options were used: --length-sigma 1 --plot-fit 3 --min-k 17 --max-k 41 --sketch-size 100000. Cluster fitting was performed using dbscan with a --K of 6 and the output from that then refined. The output of this refinement was used to produce a neighbor-joining tree, which was uploaded to the Interactive Tree of Life (https://itol.embl.de/) (Letunic and Bork, 2024) and modified there.
